# Pseudoexon activation increases phenotype severity in a Becker muscular dystrophy patient

**DOI:** 10.1002/mgg3.144

**Published:** 2015-04-15

**Authors:** Kane Greer, Kayla Mizzi, Emily Rice, Lukas Kuster, Roberto A Barrero, Matthew I Bellgard, Bryan J Lynch, Aileen Reghan Foley, Eoin O Rathallaigh, Steve D Wilton, Sue Fletcher

**Affiliations:** 1 Centre for Comparative Genomics, Murdoch University90 South St, Murdoch, Western Australia, 6150, Australia; 2 The University of Western Australia35 Stirling Highway, Crawley, Western Australia, 6009, Australia; 3 Children’s University HospitalTemple Street, Dublin, Ireland; 4 Department of Physiotherapy, Central Remedial ClinicDublin, Ireland; 5 Western Australian Neuroscience InstituteNedlands, Western Australia, 6009, Australia

**Keywords:** Cryptic splicing, Duchenne/Becker muscular dystrophy, dystrophin, exon splicing enhancer, pseudoexon

## Abstract

We report a dystrophinopathy patient with an in-frame deletion of *DMD* exons 45–47, and therefore a genetic diagnosis of Becker muscular dystrophy, who presented with a more severe than expected phenotype. Analysis of the patient *DMD* mRNA revealed an 82 bp pseudoexon, derived from intron 44, that disrupts the reading frame and is expected to yield a nonfunctional dystrophin. Since the sequence of the pseudoexon and canonical splice sites does not differ from the reference sequence, we concluded that the genomic rearrangement promoted recognition of the pseudoexon, causing a severe dystrophic phenotype. We characterized the deletion breakpoints and identified motifs that might influence selection of the pseudoexon. We concluded that the donor splice site was strengthened by juxtaposition of intron 47, and loss of intron 44 silencer elements, normally located downstream of the pseudoexon donor splice site, further enhanced pseudoexon selection and inclusion in the *DMD* transcript in this patient.

## Introduction

The most common mutations in the dystrophin (*DMD)* gene are deletions of one or more exons (Den Dunnen et al. 1989) and those that disrupt the open reading frame and ablate or dramatically reduce muscle dystrophin cause the fatal disease, Duchenne muscular dystrophy (DMD) (MIM #310200). In-frame deletions do not disrupt the reading frame and generally yield a shortened dystrophin of variable quality and function (Monaco et al. 1988). In these cases, patients typically present with the milder condition, Becker muscular dystrophy (BMD) (MIM #300376), and remain ambulant beyond 15 years of age, whereas DMD patients lose the ability to walk by age 12. One of the more common in-frame deletions expected to cause a BMD phenotype is that of exons 45–47 (Bushby et al. 1993).

In this study we report on a patient, identified as having a deletion of exons 45–47 and therefore a genetic diagnosis of BMD, who presented with an intermediate/severe BMD clinical phenotype and lost ambulation by the age of 15 years. Analysis of the *DMD* mRNA from the patient described here revealed that a pseudoexon of 82 nucleotides, derived from intron 44, (pseudoexon 44a) was included in the *DMD* transcript disrupting the open reading frame to yield a truncated, nonfunctional dystrophin. Since the sequence of the pseudoexon and flanking canonical splice sites are identical to the reference sequence, we concluded that the genomic deletion brought together elements promoting recognition and selection of exon 44a into the mature mRNA, causing a more severe dystrophic phenotype than would be expected from the loss of exons 45–47.

We sought to fully characterize the mutation in this patient, by mapping the deletion breakpoints and sequencing of the pseudoexon 44a and flanking regions, in order to identify motifs that might influence selection of the pseudoexon by the splicing machinery. We concluded that the pseudoexon donor splice site was strengthened by the close proximity of motifs from intron 47, and that the loss of intron 44 silencer elements normally downstream of the pseudoexon donor splice site further enhanced exon selection and inclusion in the *DMD* transcript.

The patient was reported with a deletion of dystrophin exons 45–47 (NG_012232.1(DMD):c.6439-?_6912+?del) but presented with a severe BMD/intermediate (IMD) phenotype. Dermal fibroblasts were prepared from a skin biopsy and provided by MRC Centre for Neuromuscular Diseases, Institute of Genetic Medicine, Newcastle University, Newcastle-Upon-Tyne, U.K. The study received approval from the Human Ethics Committee at Murdoch University (approval number 2013/156) and The University of Newcastle University (approval number 08/H0906/28+5). Fibroblasts were also derived from a small biopsy donated by a normal individual, with informed consent. Fibroblasts were propagated in Dulbecco’s modified Eagle medium (DMEM) (Life Technologies, Melbourne, Australia) supplemented with 15% fetal bovine serum (Serana, Bunbury, Australia) and induced into the myogenic lineage with a MyoD-expressing adenovirus, Ad5.f50.AdApt.MyoD (Native Antigen Company, Oxford, U.K.) (Lattanzi et al. 1998), at an MOI of 200 and allowed to differentiate as described previously (Fletcher et al. 2013). Total RNA was extracted from cultured cells and used as template (50 ng) in an RT-PCR amplifying across exons 43–51 (Wilton et al. 2007; Fletcher et al. 2013). This assay allows detection of various RNA processing events, including cryptic splice site activation or endogenous exon skipping in the region of the deletion.

**Figure 1:**
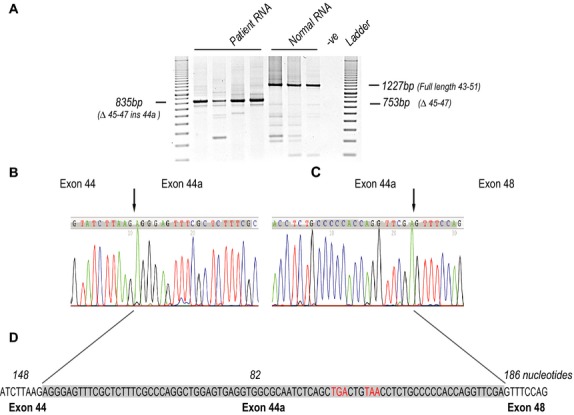
Pseudoexon identification. (A) RT-PCR amplification of normal human and *DMD* exon 45–47 deletion patient RNA samples, across dystrophin exons 43–51. Amplicon sizes are indicated, (expected size for the normal *DMD* transcript product is 1227 nucleotides, while *DMD* deletion of exons 45–47 should generate a 753 nucleotide product (*M* = 100 bp size standard ladder). The predominant transcript product amplified from patient RNA is 835 bp. Sequencing chromatograms showing the junctions of exon 44 (B) and 44a, and 44a and exon 48 (C). (D) Sequence of the 82 bp pseudoexon (exon 44a-shaded) derived from intron 44, and inserted between exons 44 and 48. The exon junctions are indicated by arrows and in-frame termination codons are shown in red. Exon sizes (bp) are indicated in italics. *DMD*, dystrophin gene.

The patient sample yielded a major product of 835 bp as well as an occasional minor product of approximate size 750 bp (Fig.1A), and the full-length product amplified from the normal RNA is of the expected size (1227 bp). It was expected that a patient with a *DMD* deletion of exons 45–47 would yield an amplicon of 753 bp, apparent as a very minor product in both patient and normal samples. Sporadic alternative splicing of the *DMD* transcript and generation of revertant transcripts has been widely reported, and transcripts missing a number of exons, including exons 45–47 are frequently observed in both normal and DMD muscle. Sequencing of the larger patient amplicon revealed the inclusion of a pseudoexon (exon 44a) of 82 bp derived from *DMD* intron 44 (NG_012232.1(DMD):c.6438_6439ins6438+192432_6438+192514) or (NG_012232.1(DMD):c.[6439-55886_6913-27860del;6438_6439ins6439-55969_6439-55888]. The sequence of exon 44a was unchanged relative to the reference sequence (NG_012232.1) that includes putative canonical acceptor and donor sites (Fig.1B–D), with splice scores of 85.36 and 71.09, respectively using the Analyzer Splice Tool (http://ibis.tau.ac.il/ssat/SpliceSiteFrame.htm) (Table1). We confirmed that the intron 44 sequence upstream of the pseudoexon location was also unchanged, and we then proceeded to locate the junction of introns 44 and 47.

**Table 1:** Potential donor splice site position, splicing motif and splice scores, as determined using the Analyzer Splice Tool (http://ibis.tau.ac.il/ssat/SpliceSiteFrame.htm), within the cryptic pseudoexon 44a sequence in normal and patient *DMD* intron 44.

Sequence position	cDNA position	Splice site type	Motif	New potential splice site	Consensus value (0–100)
(A) Normal intron 44 region around cryptic pseudoexon 44a					
30	28	Donor	GGAGTGAGG	GGAgtgagg	79.71
35	33	Donor	GAGGTGGCG	GAGgtggcg	74.79
56	54	Donor	ACTGTAACC	ACTgtaacc	69.24
76	74	Donor	CAGGTTCGA	CAGgttcga	83.84
82	80	Donor	CGAgtgatt	CGAgtgatt	71.09
108	25	Donor	gaagtacct	GAAgtacct	66.84
124	41	Donor	taggtgccc	TAGgtgccc	73.87
(B) Patient intron 44 region around cryptic pseudoexon 44a					
30	28	Donor	GGAGTGAGG	GGAgtgagg	79.71
35	33	Donor	GAGGTGGCG	GAGgtggcg	74.79
56	54	Donor	ACTGTAACC	ACTgtaacc	69.24
76	74	Donor	CAGGTTCGA	CAGgttcga	83.84
82	80	Donor	CGAgtaagt	CGAgtaagt	84.57
86	3	Donor	taagtatga	TAAgtatga	76.3
113	30	Donor	ttggtactt	TTGgtactt	72.15
135	52	Donor	caggttgaa	CAGgttgaa	72.14
141	58	Donor	gaagtgaca	GAAgtgaca	72.15
164	81	Donor	taagttaaa	TAAgttaaa	67.92

*DMD*, dystrophin gene.

**Figure 2:**
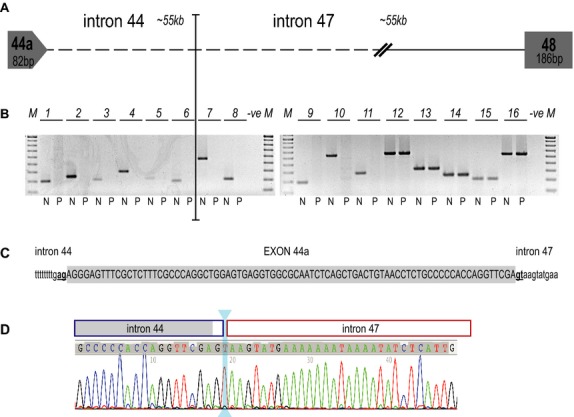
Localization of intronic breakpoints in the *DMD* exon 45–47 deletion patient. (A) Cartoon showing postulated structure of the patient dystrophin gene between pseudoexon 44a and exon 48. (B) Amplification of patient and normal DNA using primer sets designed to amplify discrete ∼350–700 bp products within *DMD* introns 44, downstream of the pseudoexon 44a (primer sets 1–6) and 47 (primer sets 7–16 [*N* = normal individual, *P* = *DMD* exon 45–47 deletion patient]). (C) Pseudoexon 44a (shaded, uppercase) and flanking intronic sequence (lowercase) showing canonical splice sites (bold, underlined). (D) Sequencing chromatogram showing the location of pseudoexon 44a (shaded) and the intron 44 (blue box) and 47 (red box) breakpoints. The origin of the “T”, two bases downstream of the 44a donor splice site remains ambiguous and is indicated by the cyan double headed arrow. *DMD*, dystrophin gene.

DNA was extracted from cultured fibroblasts, using the DNeasy Blood & Tissue kit (Qiagen, Melbourne, Australia) and used as the template in PCRs examining the *DMD* mutation and deletion breakpoints. Primer sets were designed to amplify discrete ∼350–700 bp products at ∼7 kb intervals in intron 44, downstream of the pseudoexon and at ∼5 kb intervals in intron 47 (Fig.2A) and used to amplify DNA from both the patient and a normal individual. Amplicons of the expected size were generated from both patient and normal control DNA by primer sets 12–16, whereas sets 1–11 yielded amplicons from only the normal DNA, suggesting that the breakpoint lay within intron 47, very close to the donor site of the pseudoexon (Fig.2B).

**Figure 3:**
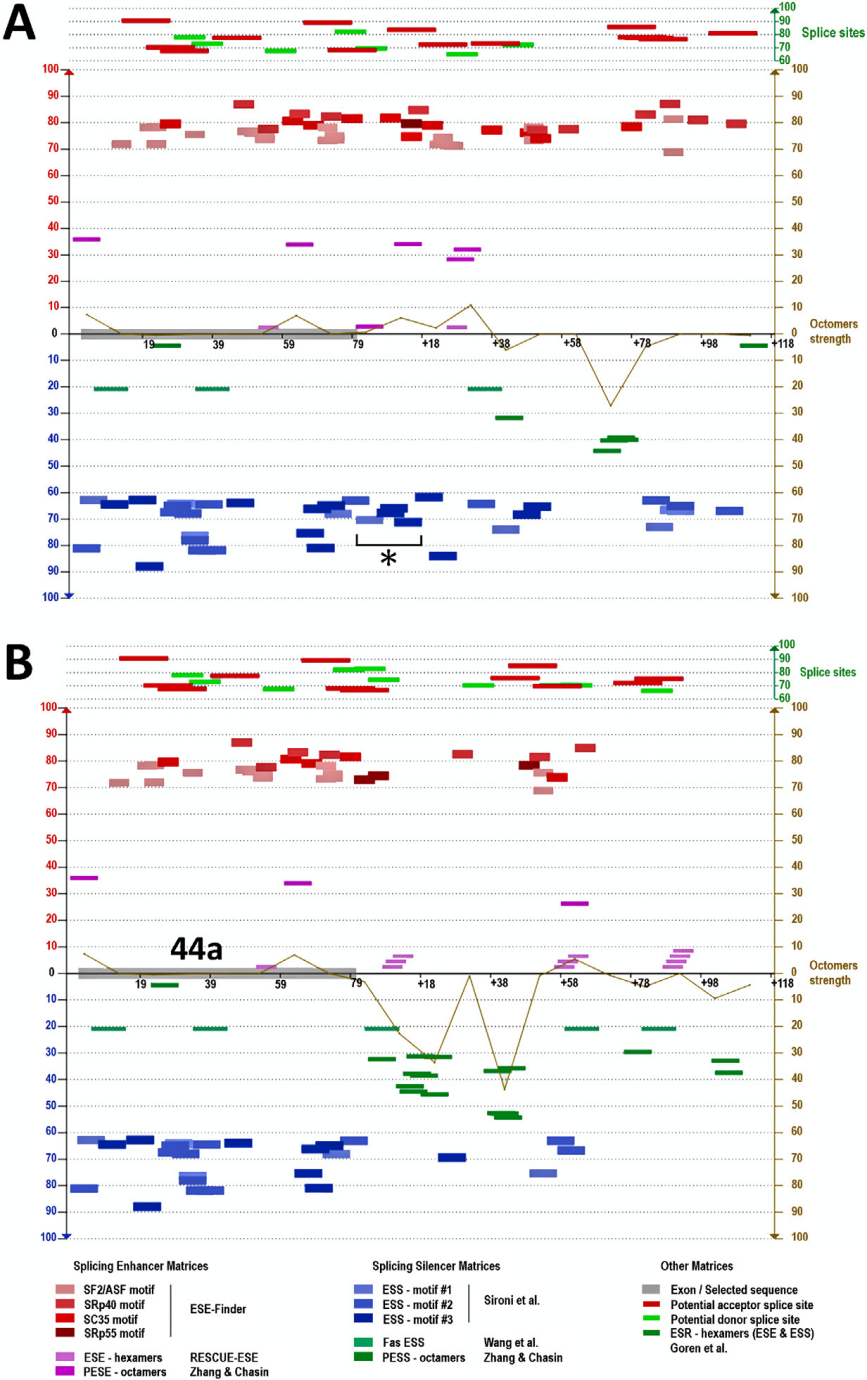
Predicted splicing motifs for the dystrophin gene pseudoexon 44a region in normal and patient DNA. (A) Predicted splicing motifs in normal intron 44 sequence including the location of 44a and downstream bases (Desmet et al. 2009). Asterisk denotes four exon splicing silencer (ESS) motifs immediately downstream of the donor splice site for 44a. (B) Predicted splicing motifs in the patient pseudoexon 44a region including downstream intron 47 bases.

In order to precisely locate the breakpoint, primers spanning the pseudoexon and the closest known sequence within intron 47 of the patient *DMD* gene were used to amplify patient DNA and each of the PCRs yielded a single amplicon (data not shown). Sequencing of the amplicons revealed that the junction of introns 44 and 47 in the patient lay one (or two) bases downstream of the cryptic donor splice site of the pseudoexon that adjoins ∼26 kb into intron 47 (Fig.2C). The juxtaposition of intron 47 and the pseudoexon at this point strengthens the donor splice site from 71.09 to 84.57 (Table1). The reference sequence shows a “*t*” at location +2 relative to the pseudoexon donor splice site in both intron 44 and intron 47 at this location (Fig.2D), so the origin of the base remains ambiguous (NG_012232.1(DMD_v001):c.6439-55886_6912+26363del,6438_6439ins6439-55969_6439-55888) or (NG_012232.1(DMD):c.[6439-55886_6913-27860del;6438_6439ins6439-55969_6439-55888]. Analysis of the *DMD* intron 44 region revealed a number of predicted splicing motifs (Desmet et al. 2009) present in the normal intron 44 sequence, including the location of exon 44a and downstream bases. Figure3 shows 4 exon splicing silencer (ESS) motifs immediately downstream of the donor splice site for 44a in the normal sequence that are missing from the patient sequence.

The patient participating in this study shows a clinical phenotype that is not consistent with deletion of *DMD* exons 45–47. He lost independent ambulation by age 15 years. His cardiac function revealed a left ventricular (LV) shortening fraction of 32% and some evidence of LV dilatation at 15 years of age, at which time he was started on a beta-blocker. At 18½ years of age he had an LV shortening fraction of 28%. The patient would be considered to have an intermediate dystrophinopathy phenotype, as he remained able to push himself in a manual wheelchair at 18½ years of age. Given the nature of his deletion, however, he would have been expected to have a classical BMD clinical presentation.

Clinical variability resulting from in-frame deletions in this region of the *DMD* gene has been widely reported. A recent study of 13 patients reported that dystrophin levels in this group varied from 13% to 76% of normal levels but did not correlate with function provided that dystrophin levels were above ∼10% of normal levels (van den Bergen et al. 2014). Similar data for in-frame deletions in this region of the *DMD* gene have been reported in earlier studies. Three patients missing *DMD* exons 45–47 had only 5% of normal muscle dystrophin levels and manifested moderate/severe dystrophy, while the other seven patients in the study, with variable mild to moderate symptoms had 20–80% of the dystrophin level found in normal healthy muscle (Kesari et al. 2008). Eleven of 43 BMD/IMD patients showed deletion of exons 45–47, with muscle dystrophin levels averaging 44% of normal (Beggs et al. 1991). Most had fairly slow progression, with four patients ranging in age from 28 to 42 remaining ambulant. However, two patients in this group were atypical, one had severe difficulty climbing stairs and the other had gross motor deficits at age 4 years and would therefore be regarded as IMD/DMD. Winnard et al. (1993) also reported a *DMD* 45–47 deletion patient as intermediate, while Dastur et al. (2008) described two of 16 patients with deletion of exons 45–47 as DMD, the remaining 14 patients are documented as BMD.

Although precise explanations for the more severe manifestation in some patients, expected to have BMD on the basis of in-frame genomic deletions, have not been reported, Muntoni and colleagues speculated that differences in the deletion breakpoints within introns could be a contributory factor (Muntoni et al. 2003). They concluded that patients with apparently identical exonic deletions are almost certainly going to have different genomic breakpoints and therefore will be missing different intronic regions, and potentially, splicing motifs. Such insight, together with the accumulating evidence of poor genotype–phenotype correlation for some patients with in-frame deletions demands more careful consideration of the nature of *DMD* genomic rearrangements.

Pseudoexon activation by altered intronic arrangements is not unprecedented. A rare intra-intronic deletion resulted in the incorporation of a segment of intron 11 as a cryptic exon into the *DMD* transcript, rendering the transcript out of frame. The aberrant transcript coexisted with a normal product in skeletal muscle, but was the only *DMD* mRNA in cardiac muscle (Ferlini et al. 1998). Individual differences in transacting splicing factors are likely to play a role in exon selection and differential dystrophin expression (Sironi et al. 2003) and deletions involving the same exons can determine diverse splicing behaviors in different patients or in different tissues of the same individual (Sironi et al. 2002).

There is currently no cure for DMD or BMD and until recently, these conditions have been managed by supportive therapies and administration of steroids (Bushby et al. 2010; Guglieri and Bushby 2010; Rodino-Klapac et al. 2013). Such therapies are beneficial for quality of life and managing comorbidities, but do not address the primary cause of the disease nor alter disease progression. Recent advances have given rise to promising potential therapies for DMD and have shown that molecular strategies can restore dystrophin in DMD muscle (Malik et al. 2010; Cirak et al. 2011; Mendell et al. 2013), for review see (Wilton and Fletcher 2013). Those that are mutation specific, such as premature termination codon read-through compounds and splice switching antisense oligomers (AOs), demand precise mutation characterization, and analysis of dystrophin expression.

AOs are single-stranded nucleic acid analogs that can be used to alter exon selection in order to restore the reading frame around a gene lesion or remove exons carrying nonsense mutations, and are showing promise in DMD (for review see Wilton and Fletcher 2011; Arechavala-Gomeza et al. 2012). The internally truncated dystrophin isoforms encoded by the manipulated transcripts contain the necessary N- and C-terminal domains that allow interaction with cytoskeletal actin and the dystrophin-associated protein complex (for review see Cohn and Campbell 2000). The production of even low levels of these BMD-like dystrophin isoforms is postulated to allow partial restoration of the dystrophin-associated protein complex, decreasing the rate of muscle fiber degeneration and conferring a degree of clinical benefit in DMD patients (Mendell et al. 2013).

To date, patients deemed to be “BMD”, on the basis of having in-frame deletions, have not been considered as candidates for exon skipping therapies. However, we suggest that reframing the dystrophin transcript in this patient by AO-mediated skipping of pseudoexon 44a could be expected to generate a functional dystrophin isoform and stabilize the dystrophic phenotype. Most patients with deletions of exons 45–47 show a mild BMD phenotype, and the minority of these patients reported to have a more severe presentation may also have more complex rearrangements or splice aberrations.

Our findings have important implications for clinical studies that aim to restore dystrophin expression in DMD patients with frame-shift deletions in this region. Rare splicing anomalies might be implicated in other genotype–phenotype inconsistencies, emphasizing the need to analyze the patient transcript in such cases. Discrepancies between genotype and phenotype should not be cited in order to undermine the predicted functionality of “BMD-like” dystrophin variants when designing exon skipping strategies to treat DMD. Such anomalous cases do not accurately reflect the clinical range for the selected patient group, if an individual’s clinical status is compromised by aberrant splicing events.
